# Chronic Intussusception Associated with Malrotation in a Child: A Variation of Waugh's Syndrome?

**DOI:** 10.1155/2016/5638451

**Published:** 2016-09-29

**Authors:** Nick Zavras, Konstantinos Tsilikas, George Vaos

**Affiliations:** ^1^Department of Paediatric Surgery, “ATTIKON” University General Hospital, School of Medicine, National and Kapodistrian University of Athens, Athens, Greece; ^2^Department of Radiology, Penteli General Children's Hospital, Athens, Greece

## Abstract

Chronic intussusception is a relatively uncommon disease most commonly observed in older children. Waugh's syndrome represents a rare entity characterized by intestinal malrotation and acute intussusception. We report a very unusual case of intestinal malrotation associated with chronic intussusception. Clinical presentation, radiological findings, and managing of this association are discussed in the light of the available literature.

## 1. Introduction

Acute intussusception is a common emergency abdominal condition in children [1]. Common clinical patterns include intermittent abdominal pain, vomiting, and “currant jelly” bloody stool [2]. Intestinal malrotation is a congenital condition caused by abnormal rotation and fixation of the bowel [3]. While bilious vomiting is the most frequent symptom in neonates, in older children intestinal malrotation is most commonly associated with nonspecific symptoms, such as chronic abdominal pain, malabsorption, diarrhea, or constipation, which may delay diagnosis [4]. The association of acute intussusception with intestinal malrotation is known as Waugh's syndrome (WS) [5]. Chronic intussusception is a distinct clinical entity, characterized by intermittent attacks of abdominal pain lasting more than 14 days; other symptoms of acute intussusception may not present. One impressive clinical feature is significant weight loss due to long-standing anorexia and vomiting [6].

Herein, we describe a rare case of chronic intussusception with intestinal malrotation that could possibly represent a variation of WS. A brief literature review of paediatric cases with WS is presented.

## 2. Case Report

**Figure 1:**
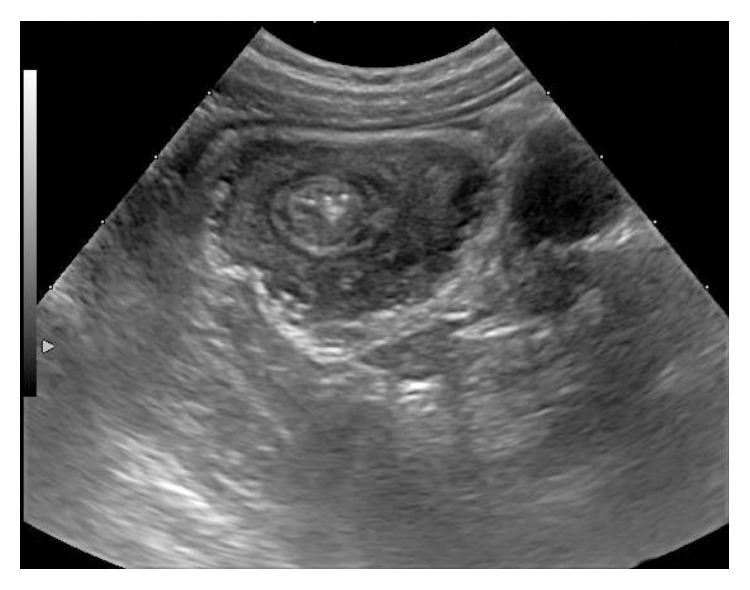
Axial U/S view of ileocolic intussusception: multiple concentric ring/donut sign.

**Figure 2:**
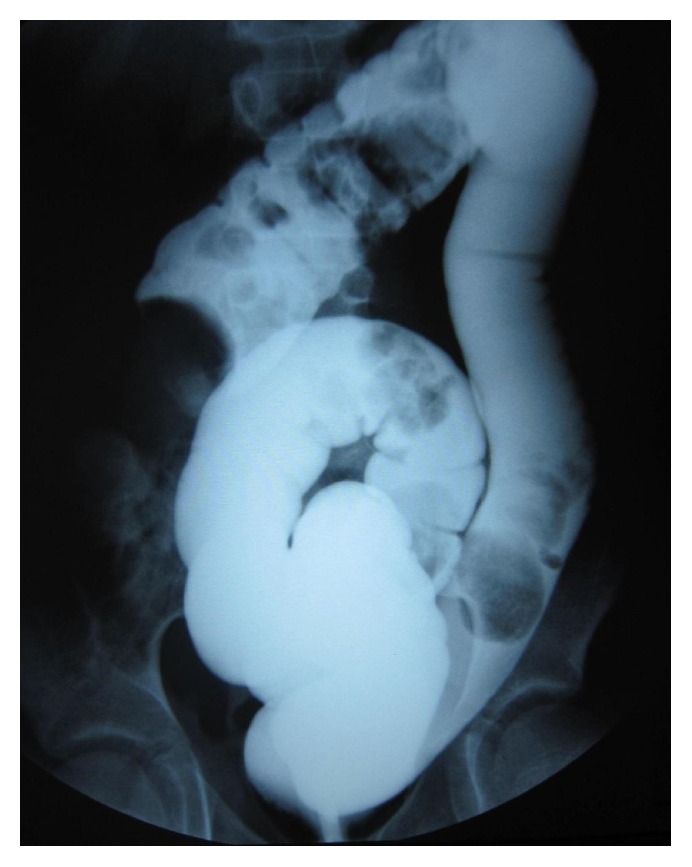
Barium contrast enema: configuration of the unsuccessfully reduced intussusception in the right upper quadrant.

A previously healthy 4.5-year-old boy was admitted to our department with a 6-week history of intermittent abdominal pain, poor appetite, sporadic nonbilious vomiting, and occasional constipation. A weight loss of five kilograms since the onset of symptoms was reported. On admission, the weight of the patient was 15 kg, as opposed to 20 kg six weeks earlier. Physical examination revealed a soft and mildly distended abdomen. A palpable, tender, round mobile mass was detected at the epigastrium. The white blood cell count was 11.000/*μ*L (normal range, 4.500–9.900/*μ*L), hemoglobin was 12.1 g/dL, and platelets were 420.000/*μ*L. The serum chemistry profile was within normal limits, apart from C-reactive protein of 0.8 mg/mL (normal range, 0–0.5 mg/mL). Abdominal ultrasonography in transverse view revealed alternating hypoechoic and hyperechoic bowel walls suggesting the target sign (Figure 1). Hydrostatic reduction was attempted, without success (Figure 2).

**Figure 3:**
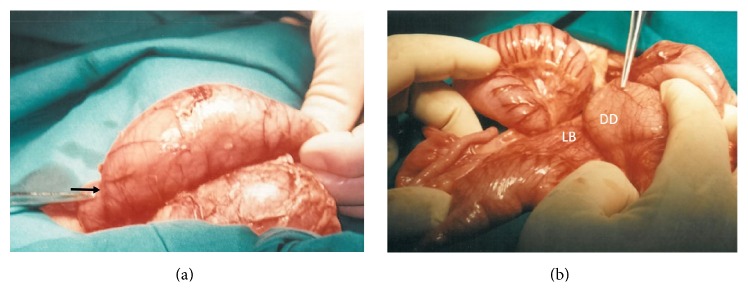
(a) Entry point of ileocolic intussusception (black arrow). (b) Ladd's bands (LB) with dilated duodenum (DD).

Exploratory laparotomy through a right upper quadrant transverse incision revealed an ileocolic intussusception extending up to the transverse colon (Figure 3(a)). The duodenojejunal junction was found to be on the right of the superior mesenteric vessels; the ileocecal junction was freely mobile and the colon was suspended by primitive mesenteric folds. Furthermore, well-defined Ladd's bands were seen to extend from the ascending colon to the posterior abdominal wall across the duodenum (Figure 3(b)).

The intussusception was manually reduced, and no leading point was found. Ladd's procedure was also performed including appendicectomy. The child had an uneventful recovery. Six months after the operation he was well without any further abdominal symptoms and had gained weight. The patient weighed 18 kg two weeks after the onset of symptoms and lost another 3 kg during the following four weeks. Six months after surgery, he weighed 22 kg (between 75th and 90th percentile).

## 3. Discussion

The incidence of chronic intussusception is about 3% of all cases of intussusception in children aged under one year and approximately 10% of children over that age [7]. The true incidence of WS is not known [5]. A PubMed and Google Scholar search revealed 33 published studies that referred to 76 children with WS (age range, 13 days to 17 years) (Table 1). To our knowledge, the association of chronic intussusception with intestinal malrotation in children has never before been mentioned in the international literature and could represent a variant of WS.

Existing evidence suggests that intestinal malrotation may predispose to acute intussusception. Waugh and Lond originally suggested that an ascending and descending colon relatively unfixed to the posterior wall and freely suspended by its primitive mesenteric folds may provoke an ileocecal intussusception [8]. According to a study by Brereton et al. [9], the principal factor that allows the terminal ileum to pass into the cecum is abnormal fixation and rotation of the ileocecal mesentery, while Breckon and Hadley [5] suggested that a mobile right colon might predispose to intussusception. In our case, one could incriminate the possible role of the freely mobile ileocecal junction as a principal factor of chronic intussusception. Moreover, the primitive mesenteric folds which do not become sufficiently tense to occlude the mesenteric blood vessels may be the cause of long-standing recurrent abdominal symptoms without any further complications such as bowel necrosis.

Ultrasonography (U/S) and contrast enema both constitute reliable imaging tools in the diagnosis of acute intussusception [1]. In the case presented herein, U/S and barium contrast enema confirmed the diagnosis of chronic intussusception. That being said, radiological evaluation was seen to offer a definite preoperative diagnosis in only seven reports of the reviewed cases of WS (Table 1, studies 12, 16, 18, 23, 25, 29, and 31).

In the majority of listed studies, open surgery (Table 1, studies 1–15, 17–22, 24, and 26–32) was the treatment of choice; a laparoscopic approach was performed in just one case (Table 1, study 25). Notably, in the studies by Breckon and Hadley [5] and Chirdan and Uba [10], intestinal malrotation was not taken into consideration during surgery for acute intussusception; hence, the patients were submitted to reoperation for recurrent symptoms of bilious vomiting. Furthermore, Lobo et al. [11] and Domingeuz-Pérez et al. [12] reported one and three cases, respectively, which were managed conservatively. In our case of a possible variant of WS, surgery was necessary to reduce the intussusception, given that the attempted hydrostatic reduction had failed. The operation had a successful outcome. All but two cases of the reviewed studies (Table 1, studies 21 and 29) were seen to have successful results.

## 4. Conclusion

The association between chronic intussusception and intestinal malrotation has never before been reported in the literature. This coexistence may represent a possible variant of WS. In cases of chronic intussusception, a high degree of suspicion is warranted in order to guide towards the proper diagnosis.

## Figures and Tables

**Table 1:** Published cases of Waugh's syndrome in the literature.

Number of studies	Author (s)	Journal	Sex	Age	Clinical onset	Type of intussusception	Type of intestinal malrotation	Outcome
1	Waugh and Lond [8]	Lancet 1911;I:1492	M (3)	2.5–3 yr	Acute	Ileocecal (2) Ileocolic (1)	Nonrotation (3)	Successful
2	Perrin	Br J Surg 1921-22;9:46	N/A	NA	Acute	N/A	N/A	N/A
3	van Meurs	Br J Surg 1946;34:91	M	5 yr	Acute	Ileocolic	Nonrotation	Successful
4	Peck	Surg Gyn Obstetr 1963;116:398	N/A	N/A	N/A	N/A	N/A	N/A
5	Tabibi	J Am Osteopath Assoc 1971;70:686	M	8 mo	Acute	Ileocecal	N/A	Successful
6	Berry	South Med J 1972;65:1075	M	17 yr	Acute	Massive ileocolic	Mobile cecum and RC	Successful
7	Stewart	Surgery 1976;79:716	N/A	N/A	Acute	N/A	N/A	Successful
8	Filston	J Pediatr Surg 1981;169 (Suppl):614	M	4.5 mo	Acute	N/A	Cecum in the RUQ	Successful
9	Ornstein	Br J Surg 1981;68:440	M	10 mo	Acute	Ileocecal	Volvulus, LB	Successful
10	Welch	Ann R Coll Surg Engl 1983;:65:244	N/A	N/A	Acute	N/A	N/A	N/A
11	Burke	Aust N Z J Surg 1985;55:73	F	3.5 mo	Acute	Ileocolic	Cecum at the level of duodenum-volvulus	Successful
12	Brereton et al. [9]	Br J Surg 1986; 73:55	N/A (15)	N/A	Acute	N/A	N/A	Successful
13	Jain	Arch Surg 1989;124:509	F M	8 mo 1.5 yr	Acute Acute	Ileocolic Ileocolic	DJJ on the right of midline, LB, volvulus	Successful Successful
14	Ward	Eur J Pediatr Surg 1992;2;239	M M	3 mo 5 yr	Acute Acute	Ileocolic Recurrent Ileocolic	IR + DS (AP) Mobile cecum, DJJ right of midline, LB (AP)	Successful Successful
15	Sarin	Indian Pediatr 1995;32:108	M	7 mo	Acute	Ileocolic	SHC	Successful
16	Lobo et al. [11]	Pediatr Radiol 1997;27:606	N/A (2)	N/A	Acute	Ileocolic (2)	MGV (1), NFAC (1)	Successful
17	Breckon and Hadley [5]	Pediatr Surg Int 2000;16;370	M (4), F (2)	4–9 mo	Acute	Ileocolic (6)	IR	Successful
18	Luo	Pediatr Surg Int 2003;19:413	M	10 mo	Acute	Ileocolic	Small bowel on the RA	Successful
19	Inan	J Pediatr Surg 2004; 39: 110	M F	8 mo 10 mo	Acute Acute	Ileocolic Ileocolic	Nonrotation IR	Successful Successful
20	Rao	Indian J Pediatr 2005;72:e21	N/A	N/A	N/A	N/A	N/A	N/A
21	Chirdan and Uba [10]	Nig J Surg Res 2005;7:159	5 M 3 F	13 d–12 mo	Acute	Ileocolic (6) Caecocolic (2)	DJJ to the right of midline (5) Cecum in RUQ (2) Volvulus + malrotation (3)	Successful (7) Died (1)
22	Lukong	S Afr J Surg 2007; 45:30	M	4 mo	Acute	Ileocolic	DJJ on the right of midline MGV	Successful
23	Rangel	Med Univers 2007;9:141	M	6 mo	Acute	Ileocolic	Malrotation, LB	Successful
24	Domingeuz-Pérez et al. [12]	Acta Pediatr Mex 2008;29:355	M (5)	2–6 mo	Acute	Ileocecal (5)	IR (5)	Successful
25	Al-Jandal	J Pediatr Surg 2009 44:E17	F	2.5 mo^*Ϯ*^	Acute	Colocolic	Nonrotation	Successful
26	Hardy	Am Surg 2011;77:78	M	3 yr	Acute	Jejunojejunal	Malrotation	Successful
27	Nwankwo	J Med Med Sci 2011;2;1291	N/A (2)	N/A	Acute	N/A	N/A	N/A
28	Baltazar	J Surg Case Rep 2012;3;22	F	3 mo	Acute	Ileocolic	DJJ to the right of SMA, LB	Successful
29	Behera	J Clin Diagn Res 2014;8:ND26	M	1 yr	Acute	Ileocolic	Nonfixed rotation	Successful
30	Al-Momami	Ann Saudi Med 2014;34:527	7 (3 M, 4 F)	4–11 mo	Acute	Ileocolic (5), ILCA (1), ILCR (1)	Malrotation (6), malrotation and volvulus (1)	Successful (6) Died (1)
31	Singh AP	J Case Rep 2014;4: 338	M	2 yr	Acute	Ileocolic	N/A	Successful
32	Natesan	J Evol Med Dent Sci 2015;4:4040	M	5 mo	Acute	Ileocolic	MGV	Successful
33	Gil-Vargas (in press)	Cir Cir 2015 (In press)	M	7 mo	Acute	Ileocolic	Abnormal fixation of the colon	Successful
34	Present case		M	4.5 yr	Chronic	Ileocolic	DJJ to the right of the midline, LB	Successful

M: male, ( ) number of patients, yr: year, N/A: not applicable, mo: month, RC: right colon, RUQ: right upper quadrant, LB: Ladd's bands, F: female, DJJ: duodenojejunal junction, IR: incomplete rotation, DS: duodenal stenosis, AP: annular pancreas, SHC: subhepatic cecum, MGV: midgut volvulus, NFAC: nonfixation of the ascending colon, RA: right abdomen, d: days, ^*Ϯ*^corrected age 38 weeks, SMA: superior mesenteric artery, ILCA: ileocoloanal, and ILCR: ileocolorectal.
